# Anthrax outbreak in Odisha, India: past, present, and future

**DOI:** 10.1097/MS9.0000000000001904

**Published:** 2024-03-11

**Authors:** Abha Deshpande, Ryan Varghese, Priyanka Roy, Mainak Bardhan, Ayush Anand

**Affiliations:** a Poona College of Pharmacy, Bharati Vidyapeeth (Deemed to be) University, Pune; b Advanced Centre for Treatment, Research, and Education in Cancer, Tata Memorial Centre, Kharghar, Navi Mumbai; c Homi Bhabha National Institute, Anushakti Nagar, Mumbai, Maharashtra; d Department of Labour, Government of West Bengal; e Indian Council of Medical Research – National Institute of Cholera and Enteric Diseases, Beleghata, Kolkata, West Bengal, India; f B. P. Koirala Institute of Health Sciences, Dharan, Nepal

*Dear Editor*,

Anthrax is a major zoonotic ailment caused by Bacillus anthracis (*B. anthracis*), a Gram-positive, spore-forming, non-motile bacterium^1^. While it mostly affects wild animals and livestock, it may spread to humans, owing to which anthrax is currently regarded as a zoonotic disease. Thus, the occurrence of the disease is substantially localized to areas involved in agronomic and livestock farming^1^. Additionally, areas with lower vaccination rates for both, humans and livestock are believed to be at a greater public health risk for Anthrax^2^. Primarily, *B. anthracis spores* are transmitted via the respiratory, gastrointestinal, or cutaneous routes. Subsequently, these spores develop into vegetative cells and proliferate within the body, ultimately precipitating symptoms such as sepsis, oedema, necrotic lesions, meningitis, and often, death^3^. Reportedly, if left unmedicated, the disease leads to increased mortality rates among humans and animals. Furthermore, anthrax has also been used as a weapon for bioterrorism^1^.

Since the year 2002, several states in India, including Andhra Pradesh, Jammu & Kashmir, Karnataka, Odisha and Tamil Nadu, have reported cases of Anthrax^2^. Especially, 14 districts of the 30 revenue districts of Odisha have experienced outbreaks of human anthrax with 1208 cases and 436 deaths over a period of 15 years leading up to 2021^4^. Of these, the districts most affected were Kandhamal, Koraput, Malkangiri, Rayagada, and Sundargarh, and accounted for more than 300 human cases and 10 deaths^5^. This can be associated with a large percentage of Odisha’s population engaged in agriculture and related occupations. Furthermore, about 22% of the total population of the state comprises indigenous tribal communities which occupy hilly terrains^5^. Moreover, various tribal practises, such as handling deskinned animal carcasses and reduced access to vaccination and medications, result in a higher incidence of Anthrax among such communities^6^. Furthermore, the poor financial conditions of these communities increase their dependence on dead animals as a food source, making them more vulnerable to exposure to anthrax^5^. Reportedly, a lack of training, accessibility, and poor adherence to protocol in medical laboratories in endemic districts, results in compromised management of the disease^5^. As these communities rely heavily on forests for their needs, they act as ‘vector bridges’^7^ for a spectrum of diseases, especially for the transmission of *B. anthracis* spores from animals to humans, leading to zoonosis^6^. Cutaneous anthrax is most commonly observed in Odisha, which manifests as a cluster of blisters, swelling, skin sores, ulcers with black centres, lesions, and oedema^6^.

Presently, in an attempt to mitigate these risks, various departments have made efforts to mediate the public health crisis associated with the recent Anthrax outbreak^8^. Currently, Bhubaneshwar’s Regional Medical Research Centre is conducting a study that spans 14 blocks of the Koraput district in Odisha, to develop and monitor the transmission of anthrax from animal to human. During the year, the study aims to include 2640 adult citizens belonging to 112 villages in the district. Various focal points of this study include widespread vaccination of animals, generating awareness among indigenous communities, and incorporating behavioural changes to reduce the risk of transmission^8^.

The current diagnostic parameters used in the detection of anthrax include the assessment of occupational exposure, the evaluation of signs and symptoms, the laboratory examination, and the microbial evaluation to detect the presence of a virulent form of the pathogen^3^. In addition, samples of biological fluids such as ascites fluid, faeces, cerebrospinal fluid (CSF), and lesions can also be tested. Samples are sought periodically to evaluate the velocity of disease progression. Management of anthrax primarily involves the use of antibiotics, such as ciprofloxacin and doxycycline, which were indicated in the cases of anthrax-mediated biowarfare^9^. Among populations considered at high risk for exposure to anthrax, prophylactic treatment may be administered to mitigate bacterial spore zoonoses^10^. A prophylactic anthrax vaccine, BioThrax, prepared from toxicogenic, non-encapsulated *B. anthracis* has reportedly demonstrated marked protection against inhaled anthrax^9^. Most importantly, post-exposure therapy relies on antibiotics, including doxycycline and ciprofloxacin, procaine penicillin G (0.6–1.2M units intramuscularly or IM every 12–24 h), and amoxicillin, which serve as the first-line drugs against occupational anthrax infections^9^. Second-line agents include drugs such as doxycycline and ciprofloxacin^10^. Later stages of infections show clinical manifestations including meningitis, sepsis, and oedema, and are often managed with drugs such as penicillin, ampicillin, ciprofloxacin, imipenem, meropenem, vancomycin, rifampin (rifampicin), clindamycin, linezolid, or aminoglycoside antibiotics^10^. Anthrax-induced meningoencephalitis is a commonly observed manifestation and is associated with an increased risk of mortality. The latter is curtailed by the administration of therapeutic interventions with marked CNS penetration including, ciprofloxacin, levofloxacin, moxifloxacin, rifampicin, and penicillin G^9^. In addition, symptomatic and supportive care is also provided, where necessary. Respiratory support in cases of compromised airways, electrolyte replacement, surgical removal of necrotic lesions, chest drainage in pulmonary infections, and thoracotomy can be imperative in severe infections^3,9^.

The roadmap to manage the disease burden in the future would benefit from identifying risk zones with optimum conditions for proliferation and survival of anthrax spores. This can aid in allocation of resources and streamline implementation of containment efforts. Lacunae in the anthrax control program in these regions can be identified to limit the spread of the disease. In this case, the management of such outbreaks is highly dependent on awareness among susceptible populations. Educating communities about the risks and caveats associated with the consumption of dead animals and the distribution of dead carcasses will prove beneficial in preventing future outbreaks. Furthermore, periodic community engagement coupled with vaccination of livestock against anthrax will also protect people engaged in animal husbandry^5,6^. Prevention of exposure, pre-exposure therapy, early detection, and rapid response are essential for proper management of the disease.

## Ethical approval

Ethics approval was not required for this letter to editor.

## Consent

Informed consent was not required for this letter to editor.

## Source of funding

The authors did not receive funding for this work.

## Author contribution

A.D. and R.V.: conceptualization, supervision, writing—original draft, writing—review and editing. M.B. and P.R.: supervision, writing—original draft, writing—review and editing. A.A.: supervision, writing—review and editing.

## Conflicts of interest disclosure

The authors have no conflict of interests to declare.

## Research registration unique identifying number (UIN)

Not applicable.

## Guarantor

Mainak Bardhan is the Guarantor.

## Data availability statement

Not applicable.

## Provenance and peer review

Not commissioned, externally peer-reviewed.
